# Work-Related Intervention Needs of Medical Assistants and How to Potentially Address Them according to Supervising General Practitioners: A Qualitative Study

**DOI:** 10.3390/ijerph19031359

**Published:** 2022-01-26

**Authors:** Jessica Scharf, Patricia Vu-Eickmann, Peter Angerer, Andreas Müller, Jürgen in der Schmitten, Adrian Loerbroks

**Affiliations:** 1 Institute of Occupational, Social and Environmental Medicine, Centre for Health and Society, Faculty of Medicine, Heinrich-Heine University of Düsseldorf, Universitätsstr. 1, 40225 Düsseldorf, Germany; jessica.scharf@uni-duesseldorf.de (J.S.); patricia.vu-eickmann@uni-duesseldorf.de (P.V.-E.); peter.angerer@uni-duesseldorf.de (P.A.); 2 Institute of Psychology, Work and Organizational Psychology, University of Duisburg-Essen, Universitätsstr. 2, 45141 Essen, Germany; andreas_mueller@uni-due.de; 3 Institute of General Practice, University of Duisburg-Essen, Pelmanstr. 81, 45131 Essen, Germany; juergen.inderschmitten@uk-essen.de

**Keywords:** medical assistants, general practitioners, work-related intervention needs, qualitative study

## Abstract

Work stress is common among health care professionals and this observation also holds true for general practitioners (GPs) and their medical assistance staff in Germany. Therefore, prior studies have examined the work-related intervention needs of medical assistants (MAs). We sought to explore potential interventions that may help to address these needs according to GPs’ views. Between December 2018 and April 2019 GPs were recruited via physician networks and through personal visits in general practices. Information on the nature and prevalence of 20 work-related intervention needs of MAs was presented to GPs. GPs then participated in a qualitative interview to reflect on the MAs’ needs. Qualitative content analysis according to Mayring was carried out using MAXQDA. A total of 21 GPs participated and perceived many of the needs as justified. The least understanding was expressed for requests of MA related to occupational aspects that were already known prior to hiring. The responsibility to address needs was often assigned to the German health policy. GPs expressed though that they considered addressing the need for better leadership style as their own responsibility as supervisors. Furthermore, professional training was discussed as one opportunity to raise the recognition and remuneration of MAs. Measures to address the work-related intervention needs of MAs and to thereby improve the working conditions of MAs were discussed with GPs.

## 1. Introduction

A broadening spectrum of treatments and responsibilities has been shifted from inpatient to outpatient care (e.g., minor surgery, disease management programs) [1,2,3] Therefore, the workload for general practitioners (GPs) is high and will likely further increase with additional tasks (e.g., gatekeeping) [4]. It has been suggested though that not only the GPs, but also their employed medical assistants (MAs) experience high levels of work-related chronic stress [5]. While there are numerous studies addressing the working conditions and work-related stress of GPs [6,7,8], the situation of MAs has only recently gained research interest [9,10,11,12].

In Germany, MAs represent the largest occupational group in outpatient care and provide basic clinical (e.g., taking blood samples or wound care) and administrative practice assistance. MAs are the first contact persons for patients and make significant contributions to patient satisfaction, patient safety and the quality of the physician-patient communication [13]. The few studies among MAs identified stressful working conditions of MAs’ such as a low salary, irregular working hours, high job demands and the need to handle unforeseeable incidents [9,14]. Work stress among health care staff, including MAs, may be associated with poor staff health (e.g., mental health) [15] and a poorer quality of patient care (e.g., medical errors) [11,14]. Furthermore, studies have also found significant associations of work stress with the intention to leave the employer or even the profession [14]. While previous studies used instruments that measure psychosocial working conditions [5,14] or job satisfaction [16], these instruments do not cover to what extent employees want or expect certain working conditions to be actually modified. For example, MAs reported that being able to perform multitasking is an essential prerequisite to work as MA and therefore inherent to the MA profession [11]. Thus, in previous studies from our group, actual work-related intervention needs have been explored based on qualitative research among MAs in Germany [11] and have been quantified in a nationwide survey [12]. The questionnaire included sociodemographic and occupational data and measured a total of 20 work-related intervention needs. In summary, 97.3% of the MAs expressed at least one need and, on average, ten needs were reported, irrespectively of sociodemographic or occupational-related characteristics [12]. The most frequently expressed needs pertained to a higher salary (87.0%), less documentation (76.0%), and more recognition from society (75.4%) [12]. Exploratory factor analysis was used to classify needs and yielded three subscales, i.e., working conditions, supervisor rewards, and task-related independence based on 12 items [12] Klicken Sie hier, um Text einzugeben. Those need subscales—in particular those pertaining to working conditions (e.g., less multitasking, more staff) and rewards from the supervisor (e.g., higher salary, understanding from supervisor)—were significantly associated with the intention to leave the employer as well as the intention to leave the profession [17], which highlights the importance to consider these needs. The fact that very few sociodemographic or occupational characteristics showed associations with the work-related intervention needs implies that there are no high-risk groups [12]. Therefore, interventions to improve their working conditions should target the entire MA population.

We sought to explore how the work-related intervention needs of MAs can be addressed in outpatient care settings from the supervisors’ perspective (GPs). This approach is promising as GPs are not only the supervisors of MAs, but also usually their employer, who has to accept and support interventions to ensure that they can be implemented effectively and sustainably. Furthermore, the GPs are likely to make particularly useful recommendations for interventions due to their detailed insights into work procedures as well as contextual factors (e.g., financial or legal aspects).

## 2. Materials and Methods

### 2.1. Study Design

We carried out qualitative interviews among GPs. Although our prior insights into work-related intervention needs relate to MAs employed mostly by either GPs or outpatient specialists [12], we focused solely on GPs to ensure that all observations relate to a similar working environment. A topic guide was used to discuss the 20 work-related intervention needs of MAs [11,12]. The work-related intervention needs were presented as statements (e.g., “I would like the GPs to have educational opportunities related to organizational leadership.”, “I would like to have a higher salary”, “I would like to have more responsibility in my job.”) with three answer categories: “Yes, I would like this”, “This need has already been met” and “No, I don’t need this”. For the interviews, the frequencies of needs of the work-related interventions were presented in tabular form which are published elsewhere [12].

The interviews were designed to span across 20 to 40 min and could either be held via telephone or in personal contact in the GP’s practice, according to the GP’s preference. There is no evidence suggesting that the themes differ by the mode of interviewing (face-to-face versus telephone interviews) [18]. A compensation of 100 € was offered to every participating GP. Interviewing was continued until thematic saturation was reached which implies that one can assume that additional interviews would not lead to the emergence of additional topics and findings [19].

### 2.2. Recruitment Process

GPs were recruited through various pathways. Firstly, GPs were contacted via an e-mail list and through quality circles from the network of outpatient GPs in Düsseldorf (Hausarztnetz Düsseldorf [HAND] e.V.). Interested GPs were asked to contact the study team by telephone or to return a signed informed consent form. Since the interviews with GPs (*n* = 10) recruited via HAND e.V. did not seem to result in thematic saturation, we continued to recruit further GPs through personal contacts as well as through personal visits in practices. In the latter case, we contacted all GP practices that were situated within randomly selected city districts of Düsseldorf. The study invitation was handed to the MAs and GPs were invited to contact the study team for participation. In total, 11 GPs were recruited by personal visits in GP practices in various districts of Düsseldorf. Furthermore, study invitations were sent via newsletters of the initiative of GPs in Bielefeld (Initiative Bielefelder Hausärzte [IBH]), however that recruitment pathway did not yield any participants. Inclusion criteria comprised the (former) management of a practice as a GP. Additionally, only GPs who employed at least one MA were included.

### 2.3. Topic Guide

We first asked the GPs to carefully review the inventory of work-related intervention needs and to express their first impression. We then asked the participants if the needs seemed reasonable and comprehensible from their point of view. Next, either all or only specific work-related intervention needs were discussed in detail, depending on the GP’s response. Based on this discussion, starting points for improvements were made the subject of discussion. The interview closed with an open-ended question for further experiences or ideas that the GP might want to share. To capture sociodemographic and practice related data, a short background questionnaire was used (see Table 1 for variables).

Our study has been approved by the Ethics Committee of the Medical Faculty of the Heinrich-Heine-University of Düsseldorf (study registration number: 2018-166-ProspeDEuA).

### 2.4. Data Collection and Analyses

Data collection was performed between December 2018 and April 2019. Interviews were digitally recorded and transcribed. All interviews were conducted and analyzed by JS who has an educational background in public health and is experienced in occupational health research [12,17] and qualitative research [20,21]. The material was analyzed using the software package MAXQDA 12 based on qualitative content analysis according to Mayring [22]. As we mainly tried to capture the views of GPs towards the intervention needs of MAs, we chose those 20 intervention needs as main categories. Based on the topic guide we further added the sub-categories: justified, responsibility and interventions. Subsequently, the coding scheme was reviewed by one co-author (PVE) and adaptations were discussed. Finally, a second coding round was conducted by JS while adaptations were made. Upon a subsequent review of the coding scheme, it was decided that two coding rounds were sufficient.

## 3. Results

### 3.1. Study Population

The study sample (Table 1) comprises 21 GPs from 20 general practices with a mean age of 56.1 years (standard deviation [SD] = 9.4) and one-third being female (*n* = 7). The average work experience is 27.6 years (SD = 8.6), while the GPs have been working in their current practice for 17.4 years (SD = 10.1) with 1.9 (SD = 0.8) GPs and 3.7 (SD = 1.5) MAs on average. Practices were all located in the same large city and treated predominantly patients with statutory health insurance. The mean number of treatment rooms was 4.8 (SD = 2.7) and documentation of patient data was mainly carried out electronically.

### 3.2. Work-Related Needs of MAs according to GPs’ View

After having reviewed all the work-related intervention needs reported by the MAs, the GPs were asked for their overall impression. It seemed that the mentioned work-related intervention needs are mostly identical with the improvements that GPs would also wish for themselves. While there are work-related intervention needs that are understandable and seem to be well justified according to GPs’ views, some needs of the MAs were more likely to be rejected or at least the responsibility for intervention was not seen to be borne by the GP. Thematically coherent types of needs that were discussed by the GPs will be presented jointly in the following. Furthermore, only the most salient ten needs of the 20 work-related intervention needs will be presented in greater details and an overview of those needs can be found in Table 2.

#### 3.2.1. Needs regarding Higher Salary and Educational Opportunities

1. Justification

GPs found MAs’ needs for a higher salary to be justified, especially when MAs work part-time. The GPs also believed that the current salary is often insufficient to feed a family and will lead to poverty in retirement (Quotation (Q 1), see online Supplementary Materials). The interviewed GPs also showed appreciation for the MAs’ tasks as MAs hold higher responsibility than many other occupational groups and therefore should be better paid accordingly (Q 2). Furthermore, GPs claimed that other trainees in the social sector earn more than MAs in training and explained that a higher degree of organization in workers’ unions would provide more political influence to raise salaries (Q 3).

GPs believed that the MA occupation has a poor reputation and therefore payment can be low which again leads to an even worse image and unattractiveness for motivated and highly qualified MAs as well as potential trainees. However, some GPs justified the (low) level of remuneration by stating that their employed MAs are contracted for 40 h a week, but most practices have opening hours of less than 40 h a week leading to an overpayment of MAs—“*so it is not all that dramatic*” (Q 4). Additionally, one GP stated the level of remuneration “*should always be seen in the context of where you live*” (Q 5) meaning that the comparatively low payment would be sufficient in a small town with lower living costs (Q 5). GPs also stated that the salary of MAs is dependent on their school education and qualification levels. These different qualification levels are already considered in the collective wage agreement so that from the GPs’ perspective no further adaptation is needed. Furthermore, the tasks of MAs were perceived to be on an assisting level while the main responsibility is always with the GP and the actual payment is thereby justified.

A related topic pertains to training opportunities, which GPs stated are widely available for MAs. The GPs also claimed that training opportunities are often offered and paid by GPs, but rejected by MAs, especially when the course takes place outside working hours (Q 6). GPs also stated that it is common to pay the training and to increase the salary of MAs after the training. Nevertheless, paid qualifications are often not utilized by the MAs in the practice.

2. Responsibility

From the GPs’ point of view, the main argument for not being responsible for increasing the salary of MAs is that they are running a commercial enterprise. As the interviewed GPs believe, profit maximization is natural in the general economy which, from the GPs’ view point, justifies that they are not responsible to share their profit (in terms of profit increases) with the MAs (Q 7). Furthermore, even if the GPs wanted to pay a higher salary, they explained that their services are state-regulated and the amount of services as well as the costs of the services are restricted under the social system. GPs therefore claimed that on a federal level the health care system is budgeted and regimented and that they also wish themselves for higher remuneration of their services. An adjustment of the doctors’ fee scale within the German statutory health insurance scheme (Einheitlicher Bewertungsmaßstab, EBM) as well as within the German private health insurance scheme (Gebührenordnung für Ärzte, GOÄ) would be necessary to firstly increase the GP earnings and to be able to then pass on the increase to the MAs (Q 8). In addition, GPs mentioned that the federal state would be responsible for introducing higher minimum wages for MAs. Pertaining to the already existing collective wage agreements, the opinions of GPs varied between seeing the need to increase the collective wage agreement and explaining that the salary of MAs has already been largely increased during the past years (Q 9). Eventually, the responsibility for the remuneration of MAs is assigned to labor unions which should exert pressure on GPs to pay higher salaries.

3. Interventions

Interventions that have already been taken by GPs and directly pertain to the remuneration of MAs is that they pay the MAs more than demanded by the collective wage agreement (Q 10). Furthermore, some GPs pay the equivalent of one monthly salary as an annual bonus and offer overtime payment. Since the monthly salary is taxable, GPs stated that MAs do not get much more payout from a salary increase. Therefore, it was common among the interviewed GPs to provide various financial or material incentives to their MAs. In Germany, a gift or voucher at a cost of 44 € per month (status from 2020) which is exempted from taxes and social security taxes, can be given to each employee (Q 11). Contributions, which have been named frequently by the GPs, pertained to the payment of public transport tickets or provision of vouchers for gas. Christmas or birthday presents have also been mentioned as common contributions as well as bearing the costs of individual health services (Individuelle Gesundheitsleistungen [IGEL]) or paying a supplementary company pension (Q 12). Some measures stated by the GPs pertained to specific circumstances or conditions such as paying a higher salary to MAs older than 40 years, since they probably will not be absent from work due to taking care of small children anymore (Q 13). Furthermore, GPs are willing to pay more, if the MA has a higher education or higher training/qualifications. Related to that, GPs see their responsibility in offering training to their MAs, but think that the responsibility to actually take part in the course lies with the MAs. Measures that GPs perceived to support MAs in completing training related to approving courses outside from working hours as working time (Q 14). GPs suggested, e.g., that the associations of statutory health insurance should reimburse only GPs (or, alternatively, reimburse them better) who pay their MAs not below the collective wage agreements, as the collective wage agreement is not binding. Nevertheless, it has been discussed by the GPs that this way of financing may lead to staff reduction (Q 15). As hiring GPs during their practical training to become a GP is subsidized in Germany, GPs also argued that the remuneration of MAs in training could be subsidized by the state (Q 16). The introduction of state regulated minimum wages for the MA profession has also been suggested. One measure considered to avoid increasing the salary is improving the working climate and to offer social activities, as this may increase the willingness to work despite low wages (Q 17).

#### 3.2.2. Needs regarding Recognition and Understanding from the Supervisor and Leadership Training

1. Justification

GPs generally responded favorably to the three presented needs pertaining to recognition and understanding from the supervisor as well as educational opportunities related to organizational leadership for the GPs. Most GPs agreed to the need for leadership trainings to improve their skills since leadership training has not been part of their medical education. GPs felt that enough training opportunities are offered. Regarding the needs for more recognition and understanding from the supervisor, the interviewees reported deficits by their GP peers, and only few thought that they could improve their expression of their recognition for their MAs. Different causes for the perceived low recognition were discussed by the interviewed GPs. One GP explained that there is a social gradient between the GPs and the MAs which leads to misunderstanding (Q 18). Furthermore, the information imbalance (e.g., medical information of the patient) may lead to the common impression of MAs that they would only act as assistant and documentation clerk (Q 19). Referring to the beginning of the career, GPs hypothesized that early career GPs may act reservedly towards MAs to cover up their insecurity and then remain in this aloofness (Q 20).

2. Responsibility

The responsibility to improve the leadership qualifications as well as recognizing the work of the MAs is clearly seen by the GPs themselves. One GP said that if the recognition is not perceived well enough by the MAs, then it is a problem of leadership (Q 21).

3. Interventions

As the interviewed GPs stated that leadership qualifications are neither part of their medical studies nor of their residency, they suggested that education in leadership should be included in their training (Q 22). Related to the GPs who are already resident doctors, the interviewees suggested supervision and leadership trainings in the practice in contrast to the currently offered leadership trainings in the form of external workshops. While some GPs could imagine spending a Wednesday afternoon on such training, (i.e., German practices are usually closed on Wednesday afternoons), other GPs stated that “*even after an eight hour training you will not become a good leader*” (Q 23). The time the interviewees would be willing to invest therefore varied between a few hours or weekends, but consensus prevailed regarding the necessity to actually use the skills in day-to-day work—especially expressing recognition and reward perishes in everyday life (Q 24). Therefore, if training is offered, GPs stated that role plays should be included to exercise the practical implementation of a rewarding working atmosphere in day-to-day work (Q 25). Furthermore, the interviewed GPs doubted that generally offered educational opportunities related to organizational leadership, which are not mandatory to practice medical care, would be utilized. Another suggestion of the interviewees pertained to the implementation of Balint-Groups, where GPs could discuss their leadership challenges and provide mutual support (Q 26). In connection with the wish for exchange with fellow GPs, the interviewed GPs suggested to implement a benchmarking system based on employee surveys among different practices. Thus, the GPs could compare the employee satisfaction and identify potentials for improvement (Q 27).

Specific measures related to the improvement of the understanding and recognition of the MAs included staff performance reviews where MAs could get feedback, but also express their concerns and may ask for work adaptations (Q 28). The GPs interpreted the need for more understanding in that way that they are responsible for finding individual solutions and to change working conditions of MAs depending on their specific situation (Q 29). Furthermore, personalized presents, such as birthday presents, bought by the GP themselves should also illustrate the interest in the person of the MA (Q 30).

#### 3.2.3. Needs regarding Multitasking and Documentation Duties

1. Justification

Most of the interviewed GPs agreed that especially the MAs working at the patient reception desk often need to carry out various tasks virtually simultaneously, if they wanted to fulfill all demands immediately. The GPs state to understand on the one hand that this demand for multitasking produces stress and may lead to errors (Q 31). On the other hand, some GPs also argued that if multitasking is overly stressful to a MA, the job as MA might just not be the right choice, as multitasking is just needed. Furthermore, one GP argued that such multitasking is one of the most interesting parts of the MA occupation (Q 32). As one time-consuming task of GPs, but also of MAs, is the documentation of patient data, both MAs and GPs wished for less documentation duties. In particular, GPs questioned that medical examinations done with electronic devices need to be separately documented (Q 33). On the contrary, one GP argued that only documented treatments can be remunerated. Furthermore, documentation of treatment is perceived important with regard to treatment errors or when legal steps are initiated (Q 34).

2. Responsibility

The GPs reported that they would also like to reduce multitasking, but they do not feel capable of achieving this. The high working demands of MAs are seen to be externally imposed by the demands of patients and are perceived to be not under control of the GP. Responsibility is therefore handed over to the patients as well as to the health care system which favors a demanding attitude, especially, among patients with a statutory health insurance. Those attitudes are supported by statements of politicians as well as the health insurance companies which assure their policyholders services that cannot be fulfilled by the GPs (Q 35). Similarly, GPs reported that they cannot reduce documentation tasks as it is a statutory requirement with regard to billing services and a burden of proof of medical treatments.

3. Interventions

While for the reduction of documentation duties only standard procedures, such as standard tables, were suggested, various measures to reduce multitasking have been mentioned. Some GPs suggested a daily structure beginning with laboratory examinations followed by consultation hours (Q 36). Additionally, consultation for acute patients were suggested to be offered around 10.30 am to 12 am, since ill patients would sleep longer and come in late. By contrast, patients who have to come for a laboratory examination and have to go to work afterwards should be able to be examined in the morning (Q 37). To reduce the number of patients at the reception desk, in some practices patients can leave their health insurance card with the MA and are sent to the waiting room (especially for data protection). Furthermore, patients were asked to order prescriptions via telephone and to pick them up the next day, instead of waiting in the practice (Q 38). Since management of telephone calls is one of the most pertinent stressors in terms of interruptions of MAs [11], most GPs wished for a separate room, where one of the MAs could answer the phone calls without any other patient contact. Some GPs had also thought about recruiting an additional part-time worker, such as a medical student, to reduce the telephone services of the MAs. A clear assignment of the MAs to work areas and tasks is seen as an important measure. Additionally, GPs found it crucial to give one assignment to only one MA and not to task all MAs with the same assignment, e.g., completing a prescription for a specific patient (Q 39). One perceived measure to reduce the multitasking are rotations to ensure that highly stressful positions (front desk) and less stressful positions (laboratory) can be switched in a certain turn (weekly, monthly etc.). Another need, expressed by MAs in our prior study [12] and the GPs in the current study, pertained to the number of present MAs. While MAs tended to wish for more staff at their workplace, GPs argued that they could not afford more staff. Relating to that measure, one GP mentioned that he closes his practice about the last two weeks of a calendar quarter. As a result, all MAs have at least seven weeks holiday a year and, apart from sick days, no MA is missing in the practice routines during the rest of the year (Q 40).

#### 3.2.4. Needs regarding Working Climate and Recognition from Society

1. Justification

GPs reported that they mainly perceive a suboptimal working climate among the MAs, rather than between the GP and the MAs (Q 41). Especially, in practices where the fluctuation among MAs is high, the working climate seems to be poor as well as in small practices where few MAs have to get along with each other. Another factor influencing the working climate is when patients are difficult or too many patients have to be treated leading to stressful working conditions of the MAs (Q 42). Some GPs also agreed that the recognition of the MAs’ work by the society/patients is low. Especially, GPs notice the difference of how (friendly) patients communicate with the GP compared to the MAs (Q 43). On the one hand, some GPs stated that the public image of the MA profession is poor, and patients also demonstrate little respect for the MAs (comparable to other social professions) which is described as an increasing problem by the GPs (Q 44). On the other hand, some GPs also reported that their MAs receive a lot of recognition (verbally or as gifts) from the patients (Q 45).

2. Responsibility

The responsibility to ensure a good working climate is seen by the GPs by themselves, since they are responsible for their staff. To protect the MAs when patients are being abusive, the GPs see the responsibility by themselves to either speak to the patient or to make the patient leave the practice (Q 46). Certainly, increasing the recognition of the MAs by the patients/society by means of public relations is not seen as part of the responsibility area of the GPs (Q 47).

3. Interventions

Pertaining to measures improving the working climate, most GPs cited the importance of team meetings among the GPs and the MAs as well as meetings only among the MAs which offer opportunities for praise and criticism. Furthermore, individual employee performance reviews offer a chance to notice the needs of the MA, which may not be discussed in team meetings (Q 48). GPs also suggested to involve the MAs in personnel decisions and to let the MAs supervise aspiring apprentices on their trial working days so that the MAs can decide if the person would fit in the team (Q 49). The interviewed GPs also highlighted the necessity to part with employees who do not fit in the team (Q 50). Joint activities, such as visiting the theatre, are mentioned as one of the most important measures to create a good working climate (Q 51). Pertaining to the recognition from the society, one of the interviewed GP thought that one should start a public campaign to increase the recognition of the MAs by the public and to increase the perceived attractiveness of the profession for apprentices (Q 52). Other than that GPs only saw the possibility to protect their MAs from rude patients, but did not know how to increase the recognition (Q 53).

In this context, the topic of patient communication arose from the interviews. Aside from higher frequencies of patient communication, such as via telephone or e-mail, the expectations of patients regarding timely appointments and treatment are very high and not attainable (Q 54). Measures to handle such difficult patients are mainly seen in deescalating measures, such as letting another MA talk to the patient or to let the GP herself/himself talk to that patient. Furthermore, GPs argued not to train MAs in handling these patients but that “the patient has to be removed from the practice” (Q 55). Other than that GPs could not image how they could change the patient communication (Q 56).

## 4. Discussion

We aimed to investigate how work-related intervention needs of MAs are perceived by GPs and how they could be addressed according to GPs, i.e., from a supervisor’s perspective. In particular the needs regarding less multitasking and higher remuneration have been seen as justified by the interviewed GPs. GPs participating in this study saw their own responsibility mainly in terms of improving their leadership skills and the working climate. The responsibility to reduce documentation duties was assigned to German health policy (i.e., legal requirements). Furthermore, the responsibility to implement interventions to increase the remuneration of MAs was allocated to the Associations of Statutory Health Insurance Physicians. Primarily, interventions pertaining to increase the MAs’ salary by further education training opportunities as well as the improved integration of leadership training in the medical studies of GPs have been discussed.

### 4.1. Findings in Light of Prior Research and Implications

Our study is the first qualitative study which specifically investigated the work-related intervention needs reported by MAs in terms of the views of supervising GPs. Based on this approach, we were able to identify measures which may address those needs and that GPs believe to be feasible and effective. These insights may guide the development and implementation of measures to actually improve the working conditions of MAs.

Since we specifically explored GPs’ attitudes towards the work-related intervention needs of MAs which have been previously examined by our group [12], the comparability of our findings to those from other studies is limited. The interviewed GPs generally focused on the wish for a higher salary, reduced documentation duties as well as reduced multitasking, which were mostly seen as justified and also said to be the needs of the GPs themselves. Pertaining to the need of an increased income, the interviewed GPs of our study reported that they see the final responsibility with themselves. Nevertheless, the GPs argued that they would first have to receive better renumeration for their medical services before they could pay their MAs a higher salary. From an economic perspective, this justification is rooted in the wage-fund doctrine [23]. That theory postulates that wages are dependent on the relative amounts of capital available for the payment of employees and the size of the labor force. Therefore, wages can only increase with an increase in capital or a decrease of the number of employees [23]. Based on this theoretical basis, the interviewed physicians would expect the Association of Statutory Health Insurance Physicians to first negotiate higher compensations with the Statutory Health Insurance Companies to pay the GPs higher compensation for each service offered. Additionally, participants also suggested that the Associations of Statutory Health Insurance Physicians should only reimburse GPs (or reimburse those better) who pay their MAs not below the collective wage agreements. Given that a higher remuneration of services should actually lead to increased revenue, it must be discussed, under the theorem of neoclassical wage theory, to what extent the revenue is used to maximize the profit of physicians or to actually increase MAs’ wages [24]. After all, the interviewed physicians also stated that they are not responsible to share their profit due to own profit maximization. The neoclassical wage theory assumes profit-maximization action by the employer and benefit-maximization by the employee [24]. Therefore, profit maximization is limited until employees can be found for the paid wages.

It has been shown that higher remuneration, in terms of recognition and salary, seems to be associated with higher job satisfaction and higher quality of care [8]. Nevertheless, wage fairness is an ongoing discussion, which needs further research. In this context, there is also evidence that even if MAs are paid according to the collective wage agreement their working conditions are still described as precarious [9]. Aside from outpatient practices, MAs can also work in hospitals. Compared to outpatient practices, MAs in hospitals are usually paid according to wage agreements and further enjoy employee rights, are protected by a work council and have better protection against dismissal [9]. In small enterprises in Germany, such as physician practices, work councils are not mandatory. Therefore, if MAs wanted to talk about improving their working conditions, they would need to contact and potentially criticize their supervisor of whom they are also financially dependent [14,17]. As a consequence, MAs most dissatisfied with their working conditions report not to pursue collective changes such as joining professional associations [9]. However, MAs primarily pursue individual-level strategies to cope with job dissatisfaction, i.e., considering leaving the employer or the profession [9,14].

Increasing recognition seems to be associated with higher job satisfaction [8] which in turn correlates with a lower intention to leave the employer or profession [14]. Therefore, one measure to promote staff retention in outpatient practices may be to increase the recognition for MAs. In this context, an intensely discussed need, for which the interviewed GPs see the responsibility with themselves, pertains to the quality of leadership and working climate. It has been shown in a systematic review that leadership quality of physicians in hospitals is associated with the retention and job satisfaction of health care staff [8]. A study among GPs in Norway found that the interviewed GPs reported a lack of knowledge concerning their leadership function [25]. Therefore, the inclusion of leadership training in career paths of GPs has been suggested, especially in terms of improved skills related to communication, motivation and conflict resolution [25]. In the present study, the interviewed GPs mainly reported that they do not feel sufficiently prepared for their leadership tasks. In Germany, the Federal Medical Association (“Bundesärztekammer”) developed a curriculum covering medical leadership, which is not mandatory, but addresses medical professionals from inpatient and outpatient care in leadership positions. Physicians in Germany are obliged to participate in ongoing professional training to the extent necessary to maintain and develop the specialist knowledge required for their professional practice. By participating in the curriculum covering medical leadership, 80 credit points of the 250 required credit points per year can be received as part of this compulsory training. As the willingness to invest time in leadership training in our study population varied between a few hours to several days, an appropriate organizational arrangement of such trainings seems to be difficult. A systematic review, mainly of studies among GPs from the USA, also found that leadership training offered by different education providers is structured differently in terms of time frame, teaching methods, educational content and transfer of knowledge [26]. Nevertheless, effective interventions are characterized by the use of multiple learning methods, such as lectures, group work as well as role play in multidisciplinary teams [26]. In summary, appropriate leadership training, such as programs that integrate GPs as well as other health professions, should be developed and offered to improve the job satisfaction of GPs and their employees. One possible way to guarantee the imparting of leadership skill for physicians may be the integration within the mandatory curriculum of medical studies. While the integration of leadership training for medical students is seen in various countries [27,28], different barriers for the implementation of leadership training in the undergraduate medical curriculum have been identified [29]. Especially, time-restriction within in current curriculum may hinder the implementation of the topic [29,30,31]. Further research is needed pertaining to the topics and methods of the leadership training as well as the implementation in the varying undergraduate medical curricula.

Another possible way to increase the recognition as well as salary of MAs pertains to the training of MAs, which enables them to take over tasks of the GPs within a legal framework. Depending on the specific training, GPs can settle up additional rates for the services performed by the trained MA. Furthermore, through delegation of tasks, GPs can offer and settle up additional services during their saved time. It has been calculated elsewhere [32] that the training and employment of a further trained MA amortized after 18 months. However, the interviewed GPs reported that they would not perceive much interest among (especially younger) MAs to participate in additional training. Participation in training may be dependent on the perception of organizational justice and the individual perception of training benefits [33]. Decisions to engage in educational opportunities (or not) can be framed in terms of the human capital theory [34]. This theory states that a person’s economic and social success is largely determined by his or her talents and abilities, which are (co-)generated through education and are developed into competencies and skills [34]. Human capital theory is based on the investment hypothesis, according to which educational activities are investments that, on the one hand, generate short-term cost (e.g., training costs, time expenditure) and, on the other hand, generate future returns or benefits (e.g., higher income, improved career prospects or skills) [34]. Therefore, it may be important to emphasize the potential long-term benefit that training may provide to MAs. The interviewed physicians felt that, overall, they express little interest in continuing education. This may be partly explained by the fact that a high proportion of MAs have a migration background (i.e., about one third of the MA population) [35]: it has been found that employees with a migration background are more likely not to participate in further education Specifically, a study among MAs showed that MAs with a migration background were more satisfied with the recognition for their work and the opportunity to use their skills compared to MAs without a migration background [16]. Therefore, the motivation for changing one’s task set or responsibilities may be rather low [16]. Reasons for the higher satisfaction could be that in particular for MAs with a migration background the completion of education and working in a team are associated with a feeling of integration, which leads to a feeling of recognition [36]. According to a large study among employees in Germany other characteristics which are associated with absence from occupational training include receiving a net income of less than 1500 € per month, working in a small company (≤50 employees), having childcare responsibilities, being female and being a younger (<24 years) or older (>55 years) employee [37]. Reasons why specifically younger MAs may tend not to participate in training according to the interviewed physicians could pertain to their work-life balance [38]. Older MAs may not participate in further training, because they do not expect further career opportunities or salary increases [39].

By contrast, prior studies suggested the wish for more responsibility and scope of action among MAs, indicating a possible misunderstanding of both parties [10,11,12]. A previous study also suggested that MAs are mainly participating in formal training because of their interest in the specific training as well as to develop professionally [10]. One established way to expand the scope of action of MAs in Germany is the possibility of (experienced) MAs, with at least two years of working experience in a GP practice, to take part in training courses as so-called (amongst others) care assistants in GPs’ practices (VERAH^®^) [10]. VERAHs are MAs who additionally monitor and coordinate services, prevention, case management and home visits on their own. Consequently, more health care services can be offered and compensated which is supposed to also increase the income of the MA, if the GPs pass the additional revenue to the MAs. Apart from income, another study found that a large degree of task delegation is positively associated with the overall job satisfaction among both GPs and their staff [40]. Moreover, employment of a VERAH in a practice has found to be associated with a reduction in hospital admissions, specialist consultations as well as medication cost [41]. These saved costs for the health care system could be (partly) reinvested in the compensation for MAs who are trained as a VERAH. The interviewed GPs of our study mainly argued that MAs lack the medical knowledge the GPs acquired during their academic studies and that MAs may increase their scope in terms of organizational tasks. However, the lack of knowledge about the legal aspects of delegating tasks has been mentioned by the interviewed GPs of our study as well in a previous study, where 39.0% of the GPs mentioned legal uncertainty [42]. Another study among GPs in Germany also reported uncertainty pertaining to legal accountability of services provided by MAs, the delegation of tasks as well as patients and relatives having problems with accepting health care services provided by VERAHs instead of the GP [43]. In contrast to the above mentioned suggestion that the services provided by VERAHs can raise the practice income and salary of the MA, the interviewed GPs reported insufficient compensation and recognition, e.g., by health insurances [43]. Studies also suggested that about three quarters of the GPs would employ a VERAH [43] and about half of the GPs have a positive attitude towards delegating tasks to non-GP staff [42]. Therefore, workshops and information sheets for GPs about employing VERAHs may help to overcome legal uncertainty regarding the delegation of tasks and to clarify how to settle up health care services provided by VERAHs.

Another issue discussed with participating GPs pertained to the reduction of executing various tasks at the same time, i.e., multitasking. Multitasking, defined as the need to do two or more tasks simultaneously [44], was perceived to be brought into the practice or to the MAs by external factors, i.e., patients, according to the interviewed GPs. Another study has analyzed the multitasking behavior of GPs and nurses and showed that interruptions from colleagues are accepted [45]. The specific reason why interruptions from others are accepted should be further analyzed to improve the communication in medical practices. In this context, specifically the demanding attitude of patients has been criticized and seen as an important measure to reduce the work demands of MAs. This is illustrated by the fact that in a previous study nearly no measures to avoid or reduce multitasking have been seen by MAs [11]. Measures suggested by the interviewed GPs mainly pertained to different consultation hours as well as restructuring the front desk of the practice. As especially patient contact via the telephone has been reported as increasing and stressful for the MAs, the participants of our study mainly discussed providing an extra room for a MA who is responsible for phone calls and will thus not be interrupted by patients in the practice. Except for spatial requirements, GPs also argued that sufficient staff would be required. In this context, some GPs suggested to recruit an assistant such as a medical student to take over telephone calls. On the one hand this might be helpful to reduce multitasking of the present MAs. On the other hand, the employment of (un-trained) assistants is mostly related to reduce employment costs (lower remuneration) compared to trained MAs for the GPs, leading to reduced available workplaces for trained MAs. A previous study among MAs showed that the MAs themselves would wish for digital information technology to schedule appointments (i.e., internet, E-Mail) in order to reduce the number of incoming phone calls [11]. As previous studies only analyzed the patients’ view of online appointments, it is to be investigated if the implementation of online appointments reduces work stress of MAs [46]. Reviewing the need “I would like to include the internet/new media in my daily work”, the interviewed GPs mainly interpreted this wish of the MAs as using the internet on their smartphone or to search for diseases online. Due to this different interpretation, the need was likely not recognized as justified or relevant. We did not clarify this different interpretation during interviewing, as we did not want to interrupt or influence the GP’s explanations.

### 4.2. Strengths and Limitations

As the study sample of GPs is only recruited from the area of the city of Düsseldorf in Germany, GPs from other geographic and structural (e.g., rural) areas are not included and their views—which may have differed—are thus not represented. However, an earlier study of GPs in Germany did not find any associations between regional characteristics and the prevalence of stress which may suggest similar issues in both urban and rural GP practices [5]. Further, we interviewed both female and male GPs, from both single and group practices, GPs with a broad range of work experience (i.e., about 19 to 36 years), and a varying number of employed MAs. This supports the notion that we were able to explore a large scope of potential views. We cannot rule out though that the topic of our study was related to the likelihood of participation. For instance, only interested and particularly committed GPs may have taken part in this study and thus we cannot exclude a selection bias, which may have restricted the scope of shared views and opinions. Additionally, in terms of social desirability, answers and opinions of the GPs may not perfectly reflect the opinions that are actually held. In this context, the GPs may have wanted to present themselves as caring supervisors and did not report difficult working situations. Furthermore, it cannot be excluded that the GPs may have used the interview politically, that is, in order to assign the responsibility for addressing many needs (e.g., a higher salary) to health policy makers and health insurances. Interviewing was carried out until thematic saturation was reached. The data has been analyzed by two independent researchers (JS and PVE) who both are experienced in performing qualitative interviews as well as analyzing interviews in the field of social and health sciences [11,20]. It might have been helpful if analysts from other professions (e.g., GPs) would have been involved in the analyzing process since they could have added another perspective. As the topic guide specifically related to justification, responsibility and potential interventions regarding the presented 20 work-related intervention needs of the MAs, data analyses were clearly focused. If we would not have focused on the specific work-related intervention needs, GPs might have referred to other topics which would not have presented the MAs needs. Nevertheless, GPs were able to suggest further relevant topics at the end of the interview.

## 5. Conclusions

Based on this qualitative study, viewpoints of GPs, who are usually both supervisors and employers of MAs, pertaining to work-related intervention needs of MAs could be explored. Discrepancies regarding the justification of MAs’ needs from the GPs’ point of view mainly arose in relation to the offer of further training opportunities for MAs as well as a desired reduction in the documentation requirement. The latter was sometimes seen as justified by the GP, but legal and billing-related requirements were seen as particularly relevant in this regard. Therefore, from the point of view of the GPs, other actors in the health care system, such as health care policy, are responsible for those needs. The GPs also stated that although they are ultimately responsible for the (improved) remuneration of the MAs, they would appreciate support from the health care policy in terms of refinancing the remuneration of their staff. In this context, further training (e.g., as VERAH^®^) was discussed as one opportunity to raise the recognition and remuneration of MAs. Interventions to improve the working conditions of MAs which were discussed with GPs mainly pertained to improving their own leadership skills. Nevertheless, very few interviewed GPs were willing to invest sufficient time in leadership training and hardly take advantage of the existing offers. One possibility could be a more extensive integration of training in leadership skills in medical studies. In summary, to improve the working conditions of MAs and to examine the practicability of various measures including those suggested by GPs, participatory intervention studies targeting the specific needs of practices are needed.

## Figures and Tables

**Table 1:** Study sample (*n* = 21).

Characteristics	Data
Age, mean (SD *)	56.1 (9.4)
Female, *n* (%)	7 (33.3)
Years in job, mean (SD *)	27.6 (8.6)
Years in current practice, mean (SD)	17.4 (10.1)
Number of GPs in practice, mean (*n*)	1.9 (0.8)
Number of GP assistants in practice, mean (*n*)	3.7 (1.5)
Number of treatment rooms, mean (SD)	4.8 (2.7)
Predominant patients with statutory health insurance, *n* (%)	21 (100)
Documentation of patient data, *n* (%)	
electronic	17 (81.0)
paper-based	1 (4.8)
both possible	3 (14.2)
Practice located in the city of Duesseldorf, Germany, *n* (%)	21 (100)
Recruitment pathway, *n* (%)	
Network of GPs in Duesseldorf (HAND e.V.)	10 (47.6)
Personal practice visits	5 (23.8)
Private contacts	6 (28.6)

* SD = standard deviation.

**Table 2:** Summary of work-related intervention needs of medical assistants and the corresponding views of general practitioners in terms of justification, responsibility and potential interventions.

Work-Related Intervention Need	Justification of the Need	Responsibility to Address the Need	Potential Interventions
**Higher salary**	MAs earn not enough to make a living on their own (i.e., without a partner), but remuneration is according to level of educational training,	Health care system: GPs (GP = General practitioner) first need to receive higher compensation for services.	Paying tax free incentives, providing health care services free of charge, invitations to social events. Paying higher salary for MAs above 40 years of age who would likely not get pregnant (again). Paying higher salaries for specially trained MAs. Supporting good working climate so MAs accept to work for less.
**More educational opportunities for MAs**	Not seen as justified.	MAs just have to take part in offered trainings (although i may takes place off the job).	Paying training fees. Approving training outside from work hours as working time.
**More recognition and understanding from the supervisor**	Deficits were seen with other physicians and only few physicians thought they could improve their own recognition for their MAs.	GPs	Offering performance reviews where MAs could get feedback, but also explain their concerns and may ask for work adaptations. Changing working conditions to the specific needs of the MAs.
**Leadership training for GPs**	Most physicians agreed that they would need organizational leadership training.	GPs	Taking part in leadership trainings (time to invest between a few hours and weekends), but consensus prevailed regarding the necessity to actually use the skills in day-to-day work. Initiating and visiting balint-groups.
**Less multitasking**	Especially at the front desk MAs have to carry out multiple tasks virtually simultaneously.	High working demands are externally imposed by the demands of patients and are perceived to be not under control of the GP; German health care system should reduce demands.	Daily structure: first laboratory examinations followed by consultation hours. Acute consultation hours between 10.30 and 12 a.m. as these patients will not go to work that day. Patients can leave health insurance card at the front desk and wait for their turn in the waiting room. Order prescriptions via phone call instead of waiting in the practice. Having an extra room for the MA answering the telephone. GP giving one task to only one MA instead of keeping all MAs busy with the same task. Offering workplace rotations to the MAs. Idea for vacations: closing practice about the last two weeks of a calendar quarter. Thereby all MAs have at least eight weeks holiday a year and no MA is missing in the practice during the rest of the year (no further increase of workload and potential multitasking).
**Less documentation duties**	Physicians stated that MAs do not have to document as much as GPs, and thus this need is not perceived as justified.	Documentation is fundamental for accountability for medical treatment and having evidence in case of legal steps.	Implementing the use of standard tables to simplify documentation procedures.
**Improved working climate**	Improvement potential of working climate seen among the MAs and less between the GP and the MAs.	GPs themselves	Team meetings with GPs and MAs and team meeting only among the MAs, which are open for appreciation and criticism. Individual performance reviews to share personal goals or problems confidentially. Involve MAs in staff-related decisions (e.g., recruitment process of new trainees or MAs). Offering team activities (e.g., dinner, visiting the theater).
**More recognition from society**	Physicians see that on the one hand the MA profession has a poor public image with patients having high demands towards MAs, on the other hand MAs can also receive high recognition from patients (e.g., verbally or presents).	Improving the recognition from the society is not seen by the GPs as their task.	GPs could join together and start a campaign to improve the public image and to increase the recognition from the society for MAs. Protect MAs from rude patients.
**Greater scope of tasks**	Only limited to occupational training and knowledge as GPs have to bear the main responsibility.	Scope of action is defined by occupational training and law, therefore not in the GPs’ field of responsibility.	Trained MAs can take over tasks within disease management programs. Managing patients in terms of aligning demanded prescriptions with prior prescriptions to report possible ambiguities to GP (e.g., last blister cannot be empty yet, interactions of medication). Organizational tasks, such as purchasing, updating the system’s software or vacation planning can be delegated to MAs.
**Improved practice organization**	GPs reported that needs related to working conditions that were known prior to hiring are not justified. Only the need for more personnel is seen as justified.	GPs feel responsible to provide enough staff, but first medical services have to be better compensated to invest in more staff.	Recruiting a part-time employee to support MAs during busy hours. Joining larger medical centers and sharing the costs for staff.

## Data Availability

The datasets generated and analyzed for the current study are not publicly available due to privacy concerns, but are available from the corresponding author on reasonable request.

## Supplementary Materials

The following are available online at https://www.mdpi.com/article/10.3390/ijerph19031359/s1, Table S1: Table of quotes.
