# Supplementary material for: Work-Related Intervention Needs of Medical Assistants and How to Potentially Address Them according to Supervising General Practitioners: A Qualitative Study
Source: Int J Environ Res Public Health. 2022 Jan 26;19(3):1359. doi: 10.3390/ijerph19031359 (PMC8835399; doi:10.3390/ijerph19031359)
Supplement: Supplementary file 1 [file ijerph-19-01359-s001.zip › ijerph-1523785-supplementary.pdf]

Online Supplement

Table of Quotes

Number	Quote
1	This implies that basically, they will all be poor in old age, unless they decide at some point to put a lot of money aside or to find a husband who makes a lot of money. (P7)
2	Of course, it is justified to ask for more money, because after all those people certainly have more responsibilities than someone who does a monotonous job like, for example, filling shelves or something like that. (P6)
3	I think it's embarrassing to find that entry salaries e.g. for kindergarten teachers or recently, there was an advertisement, when someone gets trained as a paramedic or an emergency medical assistant, that their entry salaries are higher than those of a newly trained MA. And kindergarten teachers, they sometimes go on strike. And then kindergartens are closed and they can exert pressure. In this respect MAs are not organized. (P3)
4	Well, I say, whenever you look at salaries you also have to look at working hours. Here, it's much less than 40 hours. Usually, practices aren't open for 40 hours. So, it's not that dramatic. (P11)
5	Well, of course that's the question, we don't have regional distribution of income, that also applies to other professions, certainly a problem in larger cities, also in Düsseldorf, where rents are high. And in rural regions you receive the same salary but it's cheaper to live there. (P11)
6	Because advanced training takes place in your spare time, on Wednesday afternoons, at the weekend, and of course that also counts as working hours, but they often go unnoticed. (P7)
7	Well I'm a company too, right? That's of course also operating under the aspect that I would like to make a profit. That's legitimate. (P1)<br><br>I don't know, I don't really feel obliged. If it was my impression that I would let them work harder and harder and keep the profits to myself, then I would be somewhat responsible to keep an eye on it. On the other hand, one has to say that in the free economy, that's common practice. (P7)
8	As a GP you can't do that because our work, EBM and GOÄ, they haven't really changed for a long time. (P18)
9	I say in my opinion doctor's fees agreed in the collective agreement are too low. (P2)<br><br>And I think in the last few years there was an appropriate increase. In the meantime, I think they earn about 40 % more than they did five years ago. (P6)

10	However, I am guided, or we are guided by collective agreements. We also pay an extra month's salary and we do make an effort to appreciate a good working atmosphere. (P1)
11	And then you also have the option, I have, every month I give each of them a voucher for 42 Euros. These are things I do in addition, right? (P6); Well, we sometimes grant voluntary allowances. For example, it is possible to provide a voucher for gasoline as an allowance that is tax-free. Or provide a ticket for public transports on top. (P3)
12	And if one them has a health issue, when she says: "I have a problem, I would like to see a doctor and I need a treatment from the IGel catalogue. I can't afford it", then we also cover the costs. (P18)
13	And then you also have to say, if I have an assistant who is middle-aged who is experienced, the worst-case scenario in a practice is always having several assistants who can get pregnant at the same time. So you would then say if I have an assistant aged 40 or 45 to 55, of whom I know that her health is stable and that she will not get pregnant, then I will increase her salary, because if I want to have such an assistant, then I need to take into account that perhaps she must live on her income. (P14)
14	Because advanced training takes place in your spare time, on Wednesday afternoons, at the weekend, and of course that also counts as working hours, but they often go unnoticed. (P9)
15	Well, this binding effect of collective agreements, one could make this even more binding, that the Association of Statutory Health Insurance Physicians only remunerates practices that pay salaries according to the collective agreement, or somehow make this a requirement. But this would probably only result in a reduction of staff. (P8)
16	When a physician hires another physician as a Senior House Officer, then there are subsidies. Granted by the state. That would be another option. (P3)
17	I need to make sure that there is a good working atmosphere. Then they will say: "Okay, I am willing to work for less money because I love my job." (P14)
18	Because physicians have a tendency to only know their own social milieu in other areas, too, unfortunately one has to make that statement, right? Well, now, and in a practice two different social milieus meet. And then there is a lack of understanding sometimes. And one should investigate, I presume, what this includes with regard to physicians not understanding or not appreciating their employees enough. (P8)
19	And the doctor's assistant probably has, well, quite a limited range of tasks, right, so that she sees herself rather as a, quote, accessory or a keeper of minutes, unquote or something like this. I think then the question arises of more appreciation or of another type of appreciation when the work is done,

	as this is now the case when she is part of the team, when she just. (P10)
20	For example, this is a problem for physicians, you know, studying takes a long time and then you're stuffed with knowledge, you get full responsibility very quickly, and you're not up to it. What does a person and a physician obviously do then? He becomes inapproachable and shows a self-assurance he doesn't have at all. So first he acts like an impostor […] but not because he doesn't want to be disturbed but because he still feels insecure. And this results in a kind of arrogant defence, a negative image of physicians. And once he knows things and has acquired a certain self-assurance, then he forgets that he no longer needs to act this way, that he could now be nice and friendly and outgoing. Now he regards this as a kind of weakness. (P16)
21	I'd say this is somewhere in the middle, well I don't know, the question of recognition and appreciation of performance or the emotional, I would say that's my job. But of course, they, too, they also have a certain responsibility when it comes to relationships. (P7)<br><br>If you don't express appreciation clearly enough, then that's a problem of leadership. (P2)
22	There is a wish to educate physicians in these fields. Well, I can say it wasn't part of my education. Neither business administration nor leadership skills. But perhaps it wouldn't be a bad thing to include this with education. (P2)
23	Well, let me say eight hours of advanced training don't provide me with good leadership skills. (P2)
24	Yes, for an afternoon. About three hours. On a Wednesday afternoon. (P3)<br><br>As I don't know which options exist, I can also, let me say, eight hours of advanced training don't provide me with good leadership skills. Doesn't work. Well, you somehow have to in daily life, and to be able to do so you have to learn it somewhere. I don't know how to improve on that afterwards. (P2)<br><br>Well, I can't imagine that I would spend a whole day or the whole afternoon of a Wednesday with achieving leadership skills. Yes, of course I've done that on a Wednesday afternoon, let's say from four to seven, yes, about three hours. But a two-day seminar or something like that, that would be too much. That's for an HR manager of a large company, he will have to attend a two-week training because there are/ but we do personnel management on the side […] (P9)
25	I think what isn't helpful is sitting there and then having to look at one slide after another, how to do things. I think you must learn from practical examples. I don't know, role play or something similar. (P13)
26	What I would find good, is something like this existed for physicians, exactly, for family doctors or GPs or generally for practitioners, something like a Balint group, if you become a psychotherapist or something like this, then

	one could exchange experiences. Because there are always conflicts, right, and every doctor has his own way of solving them. And depending on how you react to the conflict, you're not always objective. (P15)
27	It would be important to introduce benchmarking in a practice and to say I'll do employee surveys on a regular basis. And then I can compare myself with my colleagues. I think it is a good thing to confront physicians, where do I stand and what are general results. (P14)
28	And there is a regular meeting, a meeting between employer and employee, on an individual basis. That would be the place to address this. Well, this could be used if it doesn't already exist. (P5)<br><br>We do a consultation hour for worries. I.e., every two weeks I have during lunch break, because there was always someone coming, every day during lunch break someone came with a request, and then I said: Hey, let's do it as we used to, let's meet for coffee or lunch every two weeks, and during the other two weeks we'll do a consultation hour for worries so that everyone can get rid of their worries. (P18)
29	And I had an assistant with a broken leg and I told her: „Alright, stay at home". And she said: „No, I get bored at home. I have nothing to do but take care of my child. I want to work." And then she worked at the reception, put her cast foot up and made phone calls or entered data. It was her own request, mind you, I didn't put her under pressure. (P7)<br><br>Well, what's important is that there are individual solutions. We had an assistant who simply said, well had some physical ailments […] And then we granted her wish for a four-day instead of a five-day week. Such things are always important, that you take into account individual conditions. (P10)<br><br>On the other hand, I'm very understanding, so if she says: "On that day I have to go the dentist, on that day I have to go here or there." And then she goes there and is not around for half a day. And it doesn't matter at all. I don't mind. Then perhaps we don't draw blood on that day. (P12)
30	And appreciation shows in very personal things like a personal gift on occasions like birthdays or something like that. It is chosen by one of the doctors and wrapped nicely, but also this time going out for dinner. (P18)
31	That's a problem. Because you can't do everything at the same time and still make no mistakes. This may tempt you to make mistakes. That could happen to me, too. (P12)<br><br>I can understand it. Well, you have to be everywhere. You counsel a patient, then the phone rings, then the doctor wants something. This is one of the stress factors. (P14)
32	Well, it does make it interesting. If you really, if you know how to deal with it and you are the type of person who thinks that's great. If you find multitasking overwhelming, that is to say that you have to do two things at a

	time, like two patients who are talking to you at the same time and an additional phone call, if you haven't learned to structure it in a way that you don' get crazy, then this not the right job. (P4)
33	There's no doubt that I have to document diagnoses. Whether I have to document every online examination additionally, if there is already, if it's all in there, is something that can be discussed. But basically, less administration would be better. (P18)
34	In my opinion, when we document, then the documentation is always, well it's as little as possible. You can't do less. Yes, you can't do less, so if certain things, for the health insurances or for billability of certain services you have to write it down, and also because of legal reasons with regard to certain things. (P20)
35	With respect to patients, expectations and: "I want everything, and I want it right now." That's usually what we're confronted with. And that's also controlled by policies and health insurances, right, who tell insured patients with plastic cards: "You're entitled to everything, right now." And that's how they appear. (P11)
36	But there have been again and again, let me say, for example in fields, where lab results, i.e. drawing blood in the morning, for example at which time this takes place and how this affects consultation hours, that's one example. We separated it, we first did lab tests and blood tests and then started with the first consultation hour. That the assistant can concentrate on one thing and then the next. (P19)
37	We offer acute consultations from 10:30 am to 12:00 pm and not from 08:00 am. That's when healthy patients come for the check-up, they want to go to work afterwards. Anyone who is sick struggles to get out of bed at ten and arrives late anyway. So, they can come later. (P18)
38	The rule is: "Please send all patients to the waiting room, collect their cards before you do so, insurance cards, put them on the desk one after the other and work it off one by one and please call patients individually for privacy reasons. […] Or order lists. We have a phone line for prescriptions. But people often express this wish: "I want to order my things now." – "Yes, then please leave your order list, it will take a moment, there are so many things to do." And then it gets put aside, and then someone works it off. That's why we wish that things had been ordered in advance, then they are prepared, that takes a lot of time. (P18)
39	Well, this mostly refers to me. What we once discussed is, the rule is: Once you've attributed a task to one assistant, don't attribute it to three others to speed up the process. Because if four people do it at the same time, that doesn't help. It binds resources, and everybody does the same thing. Well, this is one of our major rules, and usually it works. (P18)
40	Concerning holidays, we have found a special arrangement. I have, I take more holidays, I take about two weeks of holidays per quarter. And that

	implies that they have about 36 days of holidays per year. And they are paid in full. This means, 36 days of holidays with full payment, that’s comparatively good. But of course, there’s a drawback. They have to take holidays during the same time as I do. Well, what I avoid this way is that someone is still on holiday, right, when the practice is open, in other words that it’s more stressful for those who are still there. (P7)
41	And I rarely have the impression that the working atmosphere between physician and assistant is poor. I often get the feeling, because I have also seen it here with another assistant, that they are at war with each other without end. Well, I believe that there’s more stress on the assistant level then between physician and assistant. (P15)
42	When you have difficult patients, who complain, because they don’t get in right on time. When occasionally there are many unscheduled patients, something like a flu epidemic. […] And of course, it happens everywhere occasionally, that there’s trouble or that people snub each other, one has a bad day and so on. (P3)
43	Well, I can hear it through the door how people talk to the assistants and how the patient later sits in front of me. And then everything is great, everyone is super nice. And out there it’s often unfriendly. (P13)
44	“More appreciation for my work from society”, that’s super difficult. It’s getting worse and worse, that’s what I feel. (P13)
45	I tell you, I see how my patients appreciate the assistants which is evident from the amount of gifts and money they take away at Christmas. I’ve never seen anything like this. It’s true, we speak of large sums. (P18)
46	When unsatisfied customers insult my assistants, then I think it’s my job to protect them and to say: “Look, they must always treat you nice and friendly because I told them so, now you don’t stick to it, as a consequence I have to protect them now. And if you want to complain, then I am the first one to address. Because I can defend myself any way I want. I can tell you: You’re no longer welcome in my practice. They can’t do that. Because I told them: “You must always be friendly.” But when the point is reached where they need protection, then that’s my job. (P7)
47	Well, to start with the larger context, with public relations, I myself see few opportunities. I can’t tell a patient, well, these are the best assistants ever. I think this is a general thing, how, let me say it like this, we as physicians present our employees in a larger context. (P1)
48	Well, of course we have introduced a team meeting we do every four weeks. And then we asked to introduce an assistants’ meeting, also every four weeks, with which we physicians have nothing to do, where they can solve problems together. (P18)<br><br>And the meeting with individual employees, I think it’s important, because that’s where you can talk about certain things you don’t talk about in the

	team. And I also think it's good to talk about perspectives once a year or once every six months, where you can talk about how you felt in the last six months, if there are any wishes, if you would like to change your position, if there is a wish for advanced training. (P15)
49	Well, for example when we hire a new apprentice, then she's working on a trial basis for a couple of days, and then we ask the whole team, what's your impression? How does it fit? What is your preference? Who's to be considered? They also see the applications. (P3)
50	You must also be willing to let people go. When the chemistry is not right, then there's no use in continuing to work together. Instead, you have to be willing to draw a line. So that in the end the working atmosphere in these small teams continues to be good. (P9)
51	We also always try to, well, we do a lot of things, I don't know, we go to concerts, we go to the theatre, we go out for dinner and such things. (P13)
52	Well, when they say: "I would like more recognition", be it from us or from society, that you have to think about whether we as employers should take the offensive and try to present this profession differently, so that we will also find junior staff. (P1)
53	Well, if I notice it, depending on how pronounced it is, then I also tell the patient: "Please consider how you talk to the assistants. This wasn't quite friendly." (P13)
54	Patients ask for more and more internet services, for example an appointment exchange, making appointments via the internet, that you are available via e-mail. Well, the way patients communicate is changing. It's going to change dramatically. Well, in the beginning the phone rang perhaps ten times a day. Now it's ringing ten times a minute. Because people communicate differently. (P1)
55	[…] when difficult patients are not only difficult, but also unfriendly, or show no respect or start to insult, then difficult patients must be removed from the practice. It's not the assistant who needs to receive training on how to deal with them so that things work better. Right? Patients need to be removed from the practice then. I repeat it again. (GP5)
56	We are also aware of certain de-escalation strategies. First, to hold back, to remain friendly and so on. "Of course. Of course." But sometimes it doesn't work. (GP11)<br><br>And when someone is rude to one of my assistants, then I say: "Look, I can understand that you're upset. We didn't do a good job", that can happen. Or perhaps we did a good job, whatever. "But this tone of voice doesn't work. And if you don't want to understand it, then please stop coming to me. I don't like it. If you want to come back, then please apologize to my assistant

	because you have done her wrong. And if you don't like to do that, then don't return to my practice. Except in cases of life-threatening emergencies." (GP7)<br><br>No, we don't do additional training. There are a lot of courses: How to deal with difficult patients? They're not productive. They don't help the assistants, they help the difficult patients. It's difficult patients who need to change, it's not the assistant who needs to change. And when a difficult patient is difficult, any assistant with a bit of work experience can deal with it, but when difficult patients are not only difficult, but also unfriendly, or show no respect or start to insult, then difficult patients must be removed from the practice. It's not the assistant who needs to receive training on how to deal with them so that things work better. Patients need to be removed from the practice then. I repeat it again. (GP5)<br><br>Yes, it would have to begin with patients. That's the problem. That's where it would have to begin. But, what can I do? Hang up plates: "Please always be friendly"? I don't know, well this is a general attitude towards the services sector. (GP11)
